# Anterior component separation for complex ventral hernias: outcomes of a tailored reconstruction strategy

**DOI:** 10.3389/fsurg.2026.1902755

**Published:** 2026-07-24

**Authors:** Akay Edizsoy, Kerim Karslı, Serhan Akalın, Pars Tunçyürek

**Affiliations:** Department of General Surgery, Adnan Menderes University, Aydın, Türkiye

**Keywords:** abdominal wall reconstruction, component separation, fascial closure, surgical outcomes, ventral hernia

## Abstract

**Objectives:**

To evaluate the outcomes of two open ventral hernia reconstruction strategies selected according to defect complexity, with particular emphasis on postoperative morbidity and recurrence-related reoperations.

**Methods:**

This retrospective cohort study included 266 patients who underwent elective ventral hernia repair between 2020 and 2024. Patients underwent either anterior component separation or primary fascial closure with onlay polypropylene mesh reinforcement according to defect complexity and the feasibility of achieving tension-free midline fascial closure. Postoperative complications occurring within 30 days and recurrence-related reoperations during follow-up were analyzed.

**Results:**

A total of 266 patients were included, comprising 90 patients in the component separation group and 176 patients in the primary closure group. Patients undergoing component separation had substantially larger fascial defects (median 150 cm^2^ vs. 25 cm^2^, *p* < 0.001). Operative time and intraoperative blood loss were significantly higher in the component separation group. Overall postoperative complications occurred more frequently following component separation (15.6% vs. 9.1%, *p* = 0.04), mainly because of a higher incidence of wound infection (11% vs. 4%, *p* = 0.03). Recurrence-related reoperation rates were comparable between groups (13.3% vs. 13.1%, *p* = 0.92). Median follow-up was 48 months (IQR 36–60).

**Conclusions:**

Anterior component separation was preferentially used in patients with larger and more complex ventral hernias. Although early wound morbidity was more frequent in these patients, recurrence-related reoperation rates remained comparable to those achieved with primary fascial closure and onlay mesh reinforcement. These findings support anterior component separation as an effective reconstructive option for achieving durable tension-free midline closure in selected patients with complex ventral hernias.

## Introduction

Ventral hernias are among the most frequent complications following abdominal surgery and still pose a notable clinical and technical challenge for general surgeons (1). The incidence of incisional hernias after laparotomy varies across studies, ranging between 5% and 20% after elective median laparotomy, depending on patient-related factors and surgical technique (2).

The main objectives of hernia repair are to achieve tension-free fascial closure and provide durable mesh reinforcement, as emphasized by the Ventral Hernia Working Group, which identified systemic risk factors such as diabetes, smoking, and hypoalbuminemia and established criteria for optimal repair (3). Recent reviews have underscored the importance of preoperative patient optimization to minimize morbidity and recurrence (4).

Over the past decades, several techniques have been introduced to achieve durable closure in large or complex abdominal wall defects (5). Among these, the component separation (CS) method originally described by Ramirez enables medial advancement of the rectus abdominis muscles and facilitates primary fascial closure in extensive ventral hernias (6). However, conventional anterior CS has been associated with considerable wound morbidity because it requires wide subcutaneous dissection for muscle mobilization (7). In response to these challenges, posterior and minimally invasive approaches have been developed, showing reduced wound complications and improved recovery outcomes (8).

Despite ongoing advances in abdominal wall reconstruction, the choice of repair strategy is largely determined by defect complexity and the ability to achieve tension-free fascial closure. In routine clinical practice, primary fascial closure with mesh reinforcement is generally preferred for smaller defects, whereas component separation techniques are reserved for larger and more complex abdominal wall defects (9). The complexity of ventral hernias is primarily determined by fascial defect size, anatomical location, and the feasibility of achieving durable tension-free midline closure, factors that directly influence the choice of reconstructive technique. However, data describing the real-world outcomes of these tailored reconstructive approaches remain limited. Therefore, the present study aimed to evaluate postoperative morbidity and recurrence-related reoperations following ventral hernia repair strategies selected according to defect complexity.

## Methods

This retrospective cohort study included 269 consecutive patients who underwent elective ventral hernia repair at the Department of General Surgery, Aydın Adnan Menderes University, between 2020 and 2024. Three patients who underwent sublay mesh repair were excluded because of the limited sample size and the inability to perform meaningful subgroup analyses. The final study population consisted of 266 patients.

Clinical, operative, and follow-up data were obtained from institutional electronic medical records and operative reports. The study protocol was approved by the Aydın Adnan Menderes University Clinical Research Ethics Committee (Approval No: 2025/3, Date: 09.01.2025). All procedures were conducted in accordance with the Declaration of Helsinki. The manuscript was prepared in accordance with the STROBE reporting guidelines. Written informed consent for both the surgical procedure and the use of anonymized clinical data for research purposes was obtained from all patients at the time of hospitalization.

### Inclusion and exlusion criteria

Adult patients (≥18 years) who underwent elective ventral hernia repair using either anterior Adult patients (≥18 years) who underwent elective ventral hernia repair using either anterior component separation with onlay mesh reinforcement or primary fascial closure reinforced with onlay mesh were eligible for inclusion. Only patients with complete clinical, operative, and follow-up records were included in the final analysis.

Exclusion criteria included emergency operations for strangulated or incarcerated hernias, concomitant intra-abdominal infection, bowel resection during the same procedure, contaminated surgical fields, recurrent hernias following mesh infection or removal, and follow-up shorter than 12 months.

### Surgical techniques

All patients included in the final analysis underwent open ventral hernia repair with mesh reinforcement in the onlay position. During the study period, open anterior component separation according to the classical Ramirez technique represented the standard myofascial release technique for complex ventral hernias at our institution. Posterior component separation and transversus abdominis release (TAR) were not routinely performed during the study period.

The choice of reconstructive strategy was based on fascial defect size, abdominal wall tension, and the feasibility of achieving tension-free midline fascial closure. Primary fascial closure reinforced with onlay mesh was generally preferred for smaller defects in which satisfactory tension-free approximation could be achieved. Anterior component separation was reserved for larger or more complex abdominal wall defects requiring additional medial fascial advancement to restore midline continuity.

Anterior component separation was performed according to the classical Ramirez technique. Following release of the external oblique aponeurosis, medial advancement of the rectus muscle complex allowed primary midline fascial closure, followed by onlay mesh reinforcement.

In the non-component separation group, primary fascial closure was achieved without myofascial release and reinforced with onlay mesh.

A standard macroporous polypropylene mesh was used for onlay reinforcement in all patients included in the study.

### Definitions and perioperative variables

Operative time was defined as the interval between skin incision and skin closure (skin-to-skin time), excluding anesthesia preparation and recovery periods.

Defect size was measured intraoperatively. Defect area was calculated in square centimeters by multiplying the maximal longitudinal and transverse diameters of the fascial defect.

Demographic and perioperative variables included age, sex, body mass index, diabetes mellitus, hypertension, chronic obstructive pulmonary disease, cardiac disease, smoking status, American Society of Anesthesiologists (ASA) classification, preoperative hemoglobin level, and serum albumin concentration.

Preoperative serum albumin was evaluated as a surrogate marker of nutritional and inflammatory status because of its established association with wound healing and postoperative morbidity.

The primary outcome was any postoperative complication occurring within 30 days after surgery. Complications included surgical site infection, seroma requiring intervention, wound dehiscence, wound necrosis, hematoma, and other surgery-related adverse events.

The secondary outcome was recurrence-related reoperation. Reoperation was defined as any surgical intervention performed for clinically and/or radiologically confirmed ventral hernia recurrence during follow-up.

Patients were routinely evaluated in the outpatient clinic during the postoperative period. Follow-up data were obtained from scheduled outpatient visits and institutional electronic medical records. Patients with less than 12 months of follow-up were excluded from the analysis.

For descriptive purposes, hernia defects were additionally categorized according to the European Hernia Society (EHS) width classification as W1 (<4 cm), W2 (4–10 cm), and W3 (>10 cm). Width categories were derived from the recorded defect dimensions available in the institutional operative database.

### Statistical analysis

Statistical analyses were performed using IBM SPSS Statistics for Windows, version 26.0 (IBM Corp., Armonk, NY, USA).

Continuous variables were expressed as mean ± standard deviation or median with interquartile range according to data distribution assessed using the Kolmogorov–Smirnov test. Categorical variables were expressed as frequencies and percentages.

Comparisons between groups were performed using Student’s t-test or Mann–Whitney U test for continuous variables and the chi-square test or Fisher’s exact test for categorical variables, as appropriate.

All statistical tests were two-sided, and a *p* value < 0.05 was considered statistically significant.

## Results

A total of 266 patients who underwent elective ventral hernia repair were included in the analysis. Of these, 90 patients (33.9%) underwent anterior component separation (CS) and 176 patients (66.1%) underwent primary fascial closure reinforced with onlay mesh (Non-CS). The overall postoperative complication rate was 11.2%, and the overall recurrence-related reoperation rate was 13.0%. According to the EHS width classification, most patients undergoing anterior component separation had W3 defects, whereas patients treated with primary fascial closure predominantly had W1–W2 defects (Table 1).

**Table 1:** Baseline characteristics of patients undergoing ventral hernia repair.

Variable	CS (*n* = 90)	Non-CS (Onlay, *n* = 176)	*p* value
Age (years, mean ± SD)	57 ± 13	56 ± 12	0.60
Male sex (%)	51%	49%	0.73
ASA score [median (IQR)]	2 [2–3]	2 [2–3]	0.64
Albumin (g/dL, mean ± SD)	3.8 ± 0.6	3.9 ± 0.5	0.42
Diabetes mellitus (%)	27%	24%	0.59
Hypertension (%)	38%	36%	0.77
COPD (%)	12%	10%	0.81
Cardiac disease (%)	10%	9%	0.87
Renal disease (%)	4%	3%	0.74
Smoking (%)	28%	31%	0.68
Steroid use (%)	5%	4%	0.77
Body Mass Index (kg/m^2^)	29.4 ± 4.8	28.9 ± 5.1	0.48
Defect size [cm^2^, median (IQR)]	150 [100–225]	25 [4–50]	< 0.001
EHS width classification			<0.001
W1	5 (5.6%)	80 (45.5%)	
W2	32 (35.6%)	83 (47.2%)	
W3	53 (58.9%)	13 (7.4%)	

Follow-up data were obtained through scheduled outpatient visits and review of institutional records. Patients with less than 12 months of follow-up were excluded from the analysis. The duration of follow-up ranged from 12 to 62 months.

**Figure 1:**
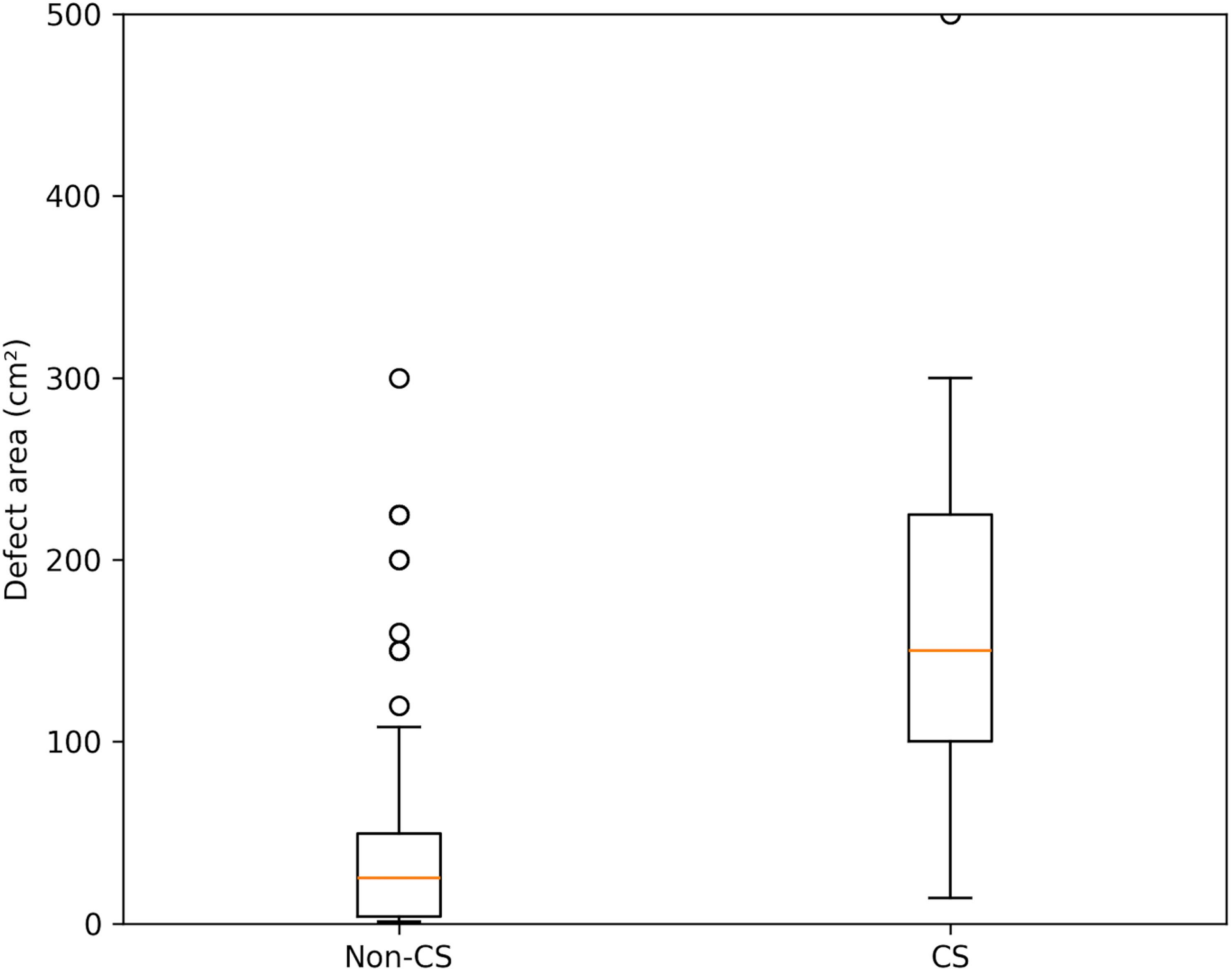
Distribution of fascial defect area according to reconstructive strategy. Boxes represent the interquartile range (IQR), the horizontal lines indicate the median, whiskers indicate the range excluding outliers, and circles represent outliers.

Baseline demographic and clinical characteristics were comparable between groups. There were no significant differences in age, sex, ASA score, body mass index, serum albumin level, or major comorbidities, including diabetes mellitus, hypertension, chronic obstructive pulmonary disease, cardiac disease, and smoking status (all *p* > 0.05). However, patients undergoing component separation had substantially larger fascial defects than those treated without component separation [median defect area 150 cm^2^ [IQR 100–225] vs. 25 cm^2^ [IQR 4–50], *p* < 0.001], reflecting the selective use of component separation in larger and more complex abdominal wall defects (Table 1). The marked difference in defect size distribution between groups is illustrated in (Figure 1).

Consistent with the greater reconstructive complexity of the component separation group, operative time and intraoperative blood loss were significantly higher than in the primary fascial closure group (159 ± 48 vs. 131 ± 37 min, *p* = 0.01; 178 ± 65 vs. 142 ± 52 mL, *p* = 0.03, respectively). Postoperative complications occurred more frequently following component separation (15.6% vs. 9.1%, *p* = 0.04). This difference was primarily driven by a higher incidence of wound infection in the CS group (11% vs. 4%, *p* = 0.03). Rates of seroma, hematoma, and wound necrosis were low in both groups (Table 2).

**Table 2:** Operative and early postoperative outcomes.

Variable	CS (*n* = 90)	Non-CS (*n* = 176)	*p* value
Operative time (min, mean ± SD)	159 ± 48	131 ± 37	0.01
Intraoperative blood loss (mL, mean ± SD)	178 ± 65	142 ± 52	0.03
Any complication (%)	15.6%	9.1%	0.04
Seroma (%)	3%	3%	1.00
Wound infection (%)	11%	4%	0.03
Hematoma (%)	1%	1%	1.00
Wound necrosis (%)	2%	0%	0.27
Recurrence-Releated Reoperation (%)	13.3%	13.1%	0.92

Despite the substantially greater defect size and operative complexity of patients undergoing anterior component separation, recurrence-related reoperation rates remained comparable between reconstructive strategies (13.3% vs. 13.1%, *p* = 0.92).

## Discussion

The present study evaluated the outcomes of two reconstructive strategies used in elective ventral hernia repair according to defect complexity. Patients undergoing anterior component separation had substantially larger fascial defects than those treated with primary fascial closure and onlay mesh reinforcement (150 cm^2^ vs. 25 cm^2^). Despite this marked difference in reconstructive complexity, recurrence-related reoperation rates were comparable between groups (13.3% vs. 13.1%). Although postoperative complications were more frequent after component separation, this increase was mainly driven by wound-related events, particularly surgical site infection. Taken together, these findings suggest that anterior component separation remains a viable reconstructive option for selected patients with complex ventral hernias.

The higher complication rate observed after anterior component separation should be interpreted in the context of defect complexity rather than surgical technique alone. In our cohort, patients undergoing component separation had defects approximately six times larger than those managed with primary fascial closure, reflecting routine surgical decision-making rather than the comparison of interchangeable procedures. Larger defects inevitably require more extensive tissue dissection and longer operations, factors known to increase wound morbidity. Consistent with this, operative time and intraoperative blood loss were significantly greater in the component separation group, indicating the higher technical demands of these reconstructions. Similar findings have been reported in national database studies and contemporary cohort analyses evaluating component separation techniques (9, 10).

The choice of reconstruction strategy in ventral hernia surgery is primarily determined by defect size and the feasibility of achieving tension-free midline closure. While primary fascial closure with mesh reinforcement is generally suitable for smaller defects, larger and more complex hernias often require myofascial advancement techniques. Since its original description by Ramirez, anterior component separation has become an established option for abdominal wall reconstruction. More recently, posterior component separation and transversus abdominis release (TAR) have expanded the available reconstructive strategies and have been associated with lower wound morbidity in selected patients (11–13). However, anterior component separation remained the standard reconstructive technique at our institution throughout the study period. Consequently, the present study reflects the outcomes of a consistent institutional approach rather than a comparison between different component separation techniques. Anterior component separation therefore continues to represent a practical and reproducible option for achieving tension-free midline closure in complex ventral hernias (14).

Wound infection was the main contributor to postoperative morbidity after anterior component separation. This finding is consistent with the wider subcutaneous dissection required to expose the external oblique aponeurosis, which has been identified as a major factor associated with wound complications following anterior component separation (15, 16). Nevertheless, the overall complication rate observed in our cohort remained within the range reported for complex ventral hernia repair (17, 18). Because all patients underwent standardized onlay reinforcement using the same macroporous polypropylene mesh, differences in postoperative outcomes are unlikely to reflect variations in prosthetic material.

One of the most important findings of this study was the comparable recurrence-related reoperation rates despite the substantially larger defects treated with anterior component separation. Hernia recurrence remains the most clinically relevant long-term outcome after abdominal wall reconstruction and reflects the durability of fascial closure. Large population-based studies have shown that recurrence continues to accumulate over time, underscoring the importance of long-term follow-up (19–21). Against this background, the similar reoperation rates observed in our cohort suggest that anterior component separation may compensate, at least in part, for the adverse effect of greater defect size by facilitating durable tension-free midline closure. Comparable findings have been reported in studies evaluating long-term outcomes after complex abdominal wall reconstruction (22).

Our findings further emphasize that outcomes after ventral hernia repair should be interpreted in the context of defect complexity rather than surgical technique alone. In routine clinical practice, reconstructive strategies are selected according to anatomical requirements and the feasibility of achieving tension-free midline closure rather than being interchangeable procedures (23). Consequently, higher early wound morbidity after anterior component separation may coexist with recurrence-related reoperation rates comparable to those of primary fascial closure despite substantially larger defects.

Several limitations should be acknowledged. First, the retrospective single-center design introduces the potential for selection bias and unmeasured confounding. Reconstruction strategy was selected according to defect characteristics and intraoperative surgical judgment rather than predefined criteria, limiting direct comparison between groups. In addition, only anterior component separation was evaluated, reflecting the institutional practice during the study period; therefore, the findings cannot be extrapolated to posterior component separation or transversus abdominis release (TAR). Follow-up, although exceeding 12 months in all included patients, was not uniform across the cohort, and recurrence was assessed by recurrence-related reoperations rather than systematic radiological surveillance, potentially underestimating asymptomatic recurrences. Finally, only open repairs were included, limiting the generalizability of our findings to minimally invasive abdominal wall reconstruction.

Despite these limitations, this study has several strengths. It reflects contemporary real-world clinical practice, includes one of the larger single-center cohorts evaluating anterior component separation, and applies a standardized reconstructive approach using onlay polypropylene mesh. Furthermore, the substantial difference in defect complexity between groups provides clinically relevant insight into the performance of anterior component separation in patients with more challenging abdominal wall defects.

## Conclusion

In conclusion, anterior component separation was primarily used in patients with larger and more complex ventral hernias. Although wound-related morbidity was more frequent in these patients, recurrence-related reoperation rates were comparable to those observed after primary fascial closure with onlay mesh reinforcement. These findings suggest that anterior component separation remains a viable reconstructive option for selected patients with complex ventral hernias.

## Data Availability

The raw data supporting the conclusions of this article will be made available by the authors, without undue reservation.
